# Stability of microRNAs in Canine Serum—A Prerequisite for Use as Biomarkers in Tumour Diagnostics

**DOI:** 10.3390/vetsci12040390

**Published:** 2025-04-21

**Authors:** Alexandra Kehl, Ruth Klein, Katja Steiger, Heike Aupperle-Lellbach

**Affiliations:** 1 Laboklin GmbH&Co. KG, 97688 Bad Kissingen, Germany; r.klein@laboklin.com (R.K.); aupperle@laboklin.com (H.A.-L.); 2 School of Medicine, Institute of Pathology, Technical University of Munich, 81675 München, Germany; katja.steiger@tum.de

**Keywords:** circulating miRNA, liquid biopsy, canine, cancer

## Abstract

Since microRNAs are released into the bloodstream and miRNA profiles are supposed to differ between healthy and tumour patients, miRNAs seem to have potential as biomarkers. An essential prerequisite for biomarkers in a routine diagnostic setup is their stability in serum over time. In this study, serum samples from 10 healthy dogs were analysed at different time points and under various temperature conditions (after 24 and 48 h, at 4° or 20 °C) for the copy number of eight miRNAs (miR-20b, 21, 122, 126, 192, 214, 222, 494) using ddPCR. The miR-21 had the highest copy number, whereas miR-494 had the lowest copy number in canine blood samples. The values of each miRNA varied individually between the dogs, showing a 5 to 10-fold range. Stability differed between the miRNAs, with miR-192 having the best stability. The copy number of miR-20b, miR-126 and miR-214 decreased not significantly during 48 h storage time. In contrast, miR-21, miR-122 and miR-222 were stable for 24 h only but decreased significantly after 48 h. The (in)stability of individual canine miRNAs must be considered when transferring study results into veterinary routine diagnostics, as the transport and storage conditions are variable. As far as possible, standardisation of sampling, storage and quantification of miRNAs is needed.

## Simple Summary

Since the levels of miRNAs differ between healthy and neoplastic patients and can be detected in the bloodstream, they are investigated as potential bio markers for cancer. To be useful in routine diagnostics, miRNAs must remain stable in blood samples over time. For stability evaluation, serum samples from 10 healthy dogs were analysed, measuring the levels of eight different miRNAs under various storage conditions (4 °C and 20 °C for 24 and 48 h). Results showed that miR-21 was the most abundant, while miR-494 was the least. The miRNA levels varied among individual dogs, sometimes by 5- to 10-fold. Some miRNAs, like miR-192, remained stable, while others, such as miR-21, miR-122, and miR-222, significantly declined after 48 h. These findings highlight the importance of considering the stability of the various miRNAs when using them as diagnostic biomarkers. Standardising miRNAs’ collection and analysis methods is essential to ensure reliable results in canine cancer detection by liquid biopsy.

## 1. Introduction

Liquid biopsy in veterinary medicine is an emerging, non-invasive diagnostic tool that detects biomarkers such as circulating tumour DNA, microRNAs, and extracellular vesicles in blood or other body fluids. It offers a less stressful alternative to traditional biopsies for diagnosing cancer, monitoring disease progression, and assessing treatment response in animals. As research advances, liquid biopsy is becoming a promising tool for enhancing veterinary oncology and precision medicine in animal healthcare [1,2].

MicroRNAs (miRNA) are increasingly used as biomarkers for cancer, heart disease, and other diseases in humans and dogs [3]. They are small, non-coding RNAs which regulate gene expression by translational suppression or enhancement [4]. They are involved in cell development and apoptosis in healthy individuals [4] and seem to play essential roles in many physiological and pathological processes, such as cancer [5]. MiRNAs can be measured in tissue but also enter the bloodstream when they are actively released by cells or during apoptosis [3,6]. They are stabilised in the blood by binding proteins or packaged in exosomes and microvesicles. These „circulating miRNAs“ can be used to diagnose and classify cancer types since each cancer entity has its unique miRNA signature [3,6]. In human medicine, many promising miRNA biomarkers are described in different tumour types, but clinical application is yet to come [7,8]. In dogs, some miRNAs are described as potential biomarkers for cancer [9,10,11,12] and liver diseases [13,14]: miR-126 and miR-214 have been identified as potential serum biomarkers for various canine epithelial cancers, as well as non-epithelial cancers [12,15]. Additionally, miR-192 and miR-20b are described as being dysregulated in intestinal carcinomas and lymphomas [9,10]. miR-214 and miR-494 showed differential expression in dogs with hemangiosarcoma and splenic hematoma compared to dogs with normal spleens [11]. Furthermore, miR-122, miR-21, and miR-222 have potential to serve as serum markers for various liver diseases [13,14].

An essential prerequisite for using miRNAs as biomarkers in routine diagnostics is their stability in liquid biopsy samples to allow shipment to a central analytical laboratory. However, until now, studies searching for differences in circulating miRNAs have not focused on their stability over time. Thus, no data are available on the optimal storage conditions of samples and storage time, and temperature differed between samples [12,13]. In contrast, several studies have investigated the stability of miRNAs in different human body fluids under various conditions (Table 1). For example, the stability of different miRNAs was investigated for a short period (up to 24 h) [16], for an extended period (miRNA isolation after 7 days) [17], or for storage at −80 °C [18,19]. In another study, EDTA blood was stored over a longer time before the extraction and isolation of miRNA, and different tube systems and centrifugation steps were compared [20]. There has been one study with canine serum only in which the samples were stored for 24 h at room temperature [21]. Regardless of the species, most studies have confirmed that serum samples provide a suitable material for miRNA isolation [22,23,24]. However, a slight or strong decline over time was observed for some or all miRNAs, resulting in noticeable differences in stability between miRNAs [16,17,18,19,20,21,23,24].

For using miRNAs as liquid biopsy markers in veterinary medicine, stability over storage must be guaranteed since analysis is regularly performed one or two days after sampling and transport to a specialised laboratory.

This study aimed to examine the stability of selected biomarker miRNAs in canine serum samples during storage at various temperatures (4 °C and 20 °C) and different points of time (2 h, 24 h, and 48 h after collection) as none of the previous studies on canine serum samples have reflected the standard conditions in veterinary routine diagnostics for these potential liquid biopsy markers.

## 2. Materials and Methods

### 2.1. Dogs and Sampling

Blood samples from 10 healthy dogs collected by local veterinarians were used in this study. These samples, taken as part of the annual check-ups, were submitted to LABOKLIN GmbH & Co. KG, Bad Kissingen, Germany, for routine diagnostic examinations. According to the terms and conditions of LABOKLIN and the decision of the government of Lower Franconia RUF-55.2.2-2532-1-86-5, no special permission has to be obtained from the animal owners or the animal welfare commission for examinations on these residual samples which were not needed for any further diagnostics.

Samples from dogs of various breeds, sexes, ages and weights were included (Table 2). According to the physical examination, the dogs were healthy, and there were no apparent clinically relevant abnormalities in the laboratory parameters. Blood was collected in serum tubes. After 30 min of incubation at room temperature, centrifugation at 1500× *g* for 10 min was performed. The supernatant was used for further processing. The copy number of miRNA was quantified by ddPCR approximately 2 h after sampling (corresponding to time point 0) and after being stored for either 24 h or 48 h at temperatures of 4 °C or 20 °C for each period.

### 2.2. RNA Isolation

Total miRNA was isolated from 200 µL serum using the miRNeasy Serum/Plasma Advanced Kit (QIAGEN, #217204, Hilden, Germany) according to the manufacturer’s instructions (miRNeasy Serum/Plasma Advanced Kit Handbook, Version April 2021). As spike-in control, cel-miR-39 was added. Total miRNA was eluted in 40 µL of RNase-free water.

### 2.3. Quantification of miRNA by ddPCR

Quantification was carried out by ddPCR using specific microRNA assays (Thermo Fisher Scientific, #4427975, Waltham, MA, USA). Reverse transcription was performed using the TaqMan^TM^ MicroRNA Reverse Transcription Kit (Thermo Fisher Scientific, #4366597, Waltham, MA, USA) according to the manufacturer’s instructions with the specific primer from the TaqMan^TM^ miRNA assay (Thermo Fisher Scientific, #4427975, Waltham, MA, USA). The ddPCR was run on the QX200 Droplet Digital System (Bio-Rad, Hercules, CA, USA) using ddPCR supermix for probes (Bio-Rad, Hercules, CA, USA) and a specific TaqMan^TM^ miRNA assay (Thermo Fisher Scientific, #4427975, Waltham, MA, USA).

Different miRNAs described as misregulated in intestinal [9,10] and splenic [25] tumours and liver diseases [13] were screened for a specific commercially available miRNA assay. TaqMan^TM^ miRNA assays were tested for their functionality in ddPCR. Finally, the following targets were chosen: miR-20b (Thermo Fisher Scientific, assay ID 001014) and miR-192 (assay ID 000493) as potential biomarkers for intestinal diseases [9,10]; miR-494 (assay ID 002365) as biomarker for splenic masses [11]; miR-122 (assay ID 002245), miR-21 (assay ID 000397), and miR-222 (assay ID 002276) as biomarkers for liver diseases such as hepatocellular carcinomas and lymphomas [13]; miR-126 (assay ID 000451) and miR-214 (assay ID 002306) as potential general biomarkers for neoplastic diseases [15]; cel-miR-39 (assay ID 000200) used for normalisation.

An amount of 10 µL cDNA was added to an 11 µL ddPCR supermix and 1 µL TaqMan^TM^ miRNA assay. The generation of droplets was carried out in 8-well cartridges: 20 µL of the abovementioned mix of cDNA, ddPCR supermix and miRNA assay was added to one well, and 70 µL oil was added to the destined well. Droplets were generated with the Droplet Maker (Bio-Rad, Hercules, CA, USA) and transferred to a 96-well plate. After sealing the plate, PCR was run with 35 cycles of 94 °C for 30 s, 60 °C for 30 s and 72 °C for 30 s. Measurements were carried out using the QX200 Droplet Reader and QuantaSoft software (Bio-Rad, Hercules, CA, USA). Each PCR was duplicated; the mean values were used for further processing. Normalisation was performed with spike-in control cel-miR-39 [26]. Inter- and intra-assay tests confirmed the validity of our test system as robust, with a variation coefficient of less than 0.15.

### 2.4. Statistical Analysis

All measurements were done in duplicate. The deviation between duplicates was used for further evaluation. The absolute miRNA copy numbers of the different time points were compared with the immediate isolation by running an ANOVA test using IBM SPSS software 29.0.1.0 for Windows. Post-hoc tests were used: Tukey’s test if homogeneity of the error variances was asserted (Levene’s test *p* > 0.05) or Games-Howell test if homogeneity of the error variances was not asserted (Levene’s test *p* < 0.05).

## 3. Results

### 3.1. miRNA Values at Time Point 0

All miRNAs analysed in this study could be detected in serum. The copy number varied individually between the dogs and the miRNAs (Table 3): At time point 0 (2 h after blood withdrawal), miR-21 was the most abundant one with a median of 63,205 copies per µL, whereas miR-494 showed the lowest value with a median of 556 copies per µL. The maximum copy number was about 10 times higher than the minimum in miR-126, 214 and 122 and about 5 times higher in miR-192, 20b, 21 and 222. There was no apparent correlation between the individual miRNA copy numbers.

### 3.2. Fold Change over Storage

The copy number of miRNA was quantified by ddPCR about 2 h after sampling (time point 0) and after storage for 24 h and 48 h at 4 °C and 20 °C. The fold change was determined by comparing the copy values to time point 0 to identify possible significant changes during transport and/or storage. A fold change of 1 means that the value is identical to the initially measured value, while 0.3 corresponds to a value of 30% of the initial value.

**Figure 1:**
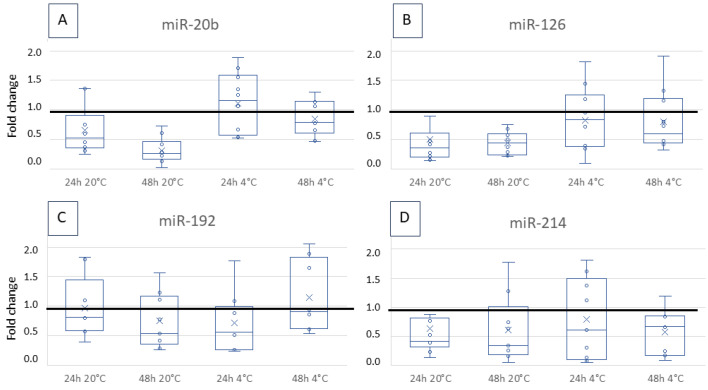
Fold change of the miR-20b (**A**), 126 (**B**), 192 (**C**) and 214 (**D**) copy number after storage for 24 h or 48 h at 4 °C or 20 °C. The changes are shown as boxplots with a median sample value and 25 per cent and 75 per cent quartiles. The copy number per µL was compared with the quantity from time point 0. The horizontal line marks a fold change of 1, which would be ideal. The variation over time and temperature was not statistically significant.

The median fold change over time and temperature was between 0.4 and 1.1 without being significant for miR-20b (Figure 1A), miR-126 (Figure 1B), miR-192 (Figure 1C), and miR-214 (Figure 1D).

**Figure 2:**
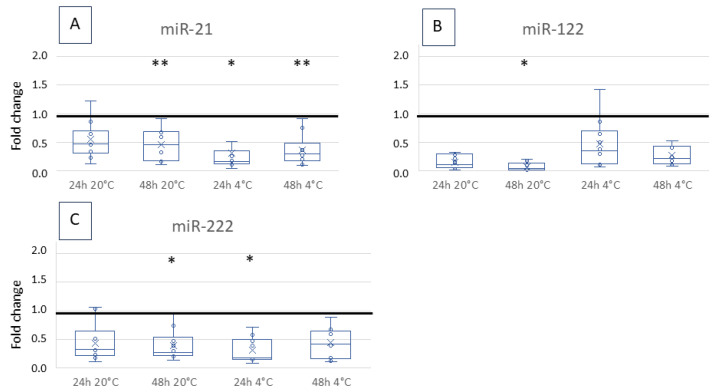
Fold change of miR-21 (**A**) 122 (**B**) and 222 (**C**) copy number after storage for 24 h or 48 h at 4 °C or 20 °C. They are shown as boxplots with a median sample value and 25 per cent and 75 per cent quartiles. The copy number per µL was compared to the quantity of time point 0. The horizontal line marks a fold change of 1, which would be ideal. Significance levels: * *p* < 0.05; ** *p* < 0.005.

In contrast, miR-21, 122, and 222 levels decreased over storage time, significantly under certain conditions (Figure 2A–C): The copy number of miR-122 declined significantly when stored at 20 °C for 48 h (*p* = 0.047). A significant decrease was also detected for miR-222 when stored at 20 °C for 48 h (*p* = 0.021) or at 4 °C for 24 h (*p* = 0.03). The copy number of miR-21 showed a significant decrease over time except when stored at 20 °C for 24 h (20 °C 48 h: *p* = 0.001, 4 °C 24 h: *p* = 0.005, 4 °C 48 h: *p* = 0.002). 

Stability tests were not performed on miRNA-494 since it was detectable but only in very low copy numbers at time point 0.

## 4. Discussion

The present study investigated the stability of different canine serum miRNAs over storage time and temperatures to evaluate the technical requirements of miRNA detection as biomarkers in routine veterinary medicine. Eight representative miRNAs were chosen: miR-126 and 214 as potential biomarkers in serum for several canine epithelial cancer types (adenocarcinomas of thyroid and mammary gland; hepatocellular, squamous cell, transitional cell carcinomas) and non-epithelial cancers (osteosarcoma, histiocytic sarcoma, chondrosarcoma, hemangiosarcoma, mast cell tumours and melanoma) [12,15]; miR-192 and 20b as being misregulated in the tissue of intestinal carcinomas and lymphomas [9,10]; miR-214 and 494 which are expressed differently in serum samples of dogs with hemangiosarcoma and splenic haematoma in comparison with normal spleens [11]; and miR-122, 21 and 222 as serum markers for different liver diseases enabling differentiation between main groups of hepatic diseases (parenchymal, vascular and neoplastic) [13]. In another study, miR-122 has been investigated more thoroughly for its usefulness as a biomarker for hepatic diseases [14]. Serum was chosen as sample material as it is a typical sample material in veterinary diagnostics, and it has already been described as suitable for miRNA quantification [22,23,24].

We detected and quantified eight miRNAs in the serum of healthy dogs, with miR-21 having the highest number of copies and miR-494 having the lowest copy number. The values within one miRNA varied individually between the dogs and showed a 5 to 10-fold interval, but without any apparent correlation to each other, indicating random variability. In healthy dogs, a wide range of miR-122 copies has already been described with 110–3312 c/µL [12] and 37–559 c/µL [25], corresponding to a variation of up-to-10-fold. In humans, even a 100-fold range within the reference interval for miR-122 was described [27]. For miR-21, 122, 126 and 222, an interval of up to 20-fold has been published in healthy control dogs [13]. Thus, copy values measured in the present study seem to be plausible despite the wide ranges.

The copy number of miR-122 in our study (231–2313 c/µL) was in the range described by Oosthuyzen et al. [14], where serum samples were immediately stored after sampling at −80 °C until RNA isolation. This confirms that ddPCR in the present study is an adequate tool to quantify this miRNA in canine serum. For the other miRNAs, no absolute copy values have been described so far. In most studies, only relative miRNA levels (compared with one or several reference miRNA levels) were investigated and/or differential expression between groups was determined by fold change analysis [11,28,29,30,31], hampering the comparability of study results.

In the present study, stability differed between the miRNAs. The miR-192 showed the highest stability with a non-significant fold change of about 0.7 to 1, independent of time and storing temperature. The decrease of miR-20b, miR-126, and miR-214 over storage time was slightly greater than for miR-192, but it was also not statistically significant. For most measurements (storage for 24 h or 48 h at 4 or 20 °C), the fold change was about 0.3 to 0.6. Interestingly, some values developed above the ideal fold change of 1, meaning more miRNA was present and/or measured than at time point 0. A reason for this could be a tolerable variation in measurement of about 10–15% [26,32,33]. In another study, increased miRNA copy numbers were found after freezing/thawing cycles, led back to possible effects of miRNAs being present unbound, or bound to proteins, or bound to extracellular vesicles [34]. Alongside other unknown effects, this could play a role here, too.

In contrast, miR-21, 122 and 222 (all potential biomarkers for liver diseases [13,14]) were extremely unstable. Some studies have already described the instability of specific miRNAs in canine and human serum. The degradation of miRNAs by RNases was mentioned as a reason for the decline over time [16,17,18]. Additionally, a distinction in stability between different miRNAs has been described for canine [21] and human samples [16,23]. The way of transport within the bloodstream may play a role in the variation of stability, with vesicle-associated miRNAs being protected from depletion compared to non-vesicle-bound miRNAs [16,23]. Furthermore, interassay precision is discussed as a cause for the differences [23]. Since we use ddPCR as a highly robust quantification technique, these problems are less critical than in qPCR. The ddPCR technique is more precise because it does not need a standard curve or an endogenous miRNA control for quantification [26,35,36]. Furthermore, sensitivity, reproducibility and accuracy of ddPCR are superior [37,38]. Even the need of replicates can be neglected [26,32]. Yet, standardisation of quantification using qPCR or ddPCR is needed.

Limitations of the present study include the small number of samples. Therefore, influence of breed, age or sex on the miRNA copy values could not be confirmed or excluded which could be a next step in validation of miRNAs as biomarkers. Furthermore, statistical power could be improved by investigating more samples. Additionally, further storage conditions and time points could be taken into consideration. Future investigations should be extended to the analysis of pathological samples in addition to healthy control samples. Last but not least, availability of reference values for miRNAs should be determined as it was done for miR-122 [14].

The instability of some miRNAs and the differences in stability described in the present and in other studies [13,18,20] must be considered when designing studies and transferring research results to routine diagnostics. In most studies, miRNA isolation was performed immediately after sampling or storage at −80 °C and degradation over time was not investigated. For miR-122, reference value for healthy (non-liver disease) dogs lies under 3312 copies per µL and mean of miR-122 copy number in liver-diseased dogs lies about 11,332 copies per µL, measured all immediately after sampling or cooling at −80 °C [14]. Taken into account that the value of miR-122 in serum after 48 h without cooling is about 10% of the value at time point 0, false negative results cannot be ruled out. Besides, a decline of 50% after 24 h cooled storage would be tolerable. In most cases, miRNAs are up-regulated when being described as potential biomarkers: miR-21, 122, 126 and 222 showed increased serum copy numbers in the liver case group than the control group [13], copy number of circulating miR-214 and 494 was raised in the group with splenic tumours [11] and miR-126 and 214 are named as biomarkers for several neoplastic diseases having an increased copy number in serum [12]. If increase of copy numbers for a certain miRNA in case group is observed, this miRNA can only be used as biomarker when fold change due to disease is remarkably more than the fold change caused by storage over time. Therefore, when using research results in routine diagnostics, the stability of miRNAs over time should be confirmed as being stable. Alternatively, the study design can include this fact, quantify the miRNAs after the usual storage time in daily routine diagnostics, and compare groups (cases and controls) with these values.

For routine diagnostics, sample material should always be the same, isolation of miRNA should be performed as fast as possible after sampling and in the same time range as done in the evaluation study, quantification should be standardized by normalization procedures. In human medicine, AI-driven miRNA profiling is increasingly investigated for cancer detection [39] presenting a promising opportunity for veterinary medicine as well.

## 5. Conclusions

Using miRNAs as biomarkers in liquid biopsy for cancer detection is becoming increasingly important, and stability over time and storage are pre-requisites. Stability varies depending on time and temperature and even between different miRNAs and species. Canine miR-20b, miR-126, miR-192, and miR-214 are stable for up to 48 h independent of temperature, whereby refrigeration likely optimises the values. While storing miR-21, miR-122, and miR-222 for 24 h at 20 °C appears satisfactory, it is essential to consistently examine the differences between control and patient samples to ensure their viability as biomarkers. These issues should be considered when designing and comparing studies and when transferring results from scientific studies to diagnostic tests.

## Figures and Tables

**Table 1:** Summary of miRNA stability studies showing investigation of different materials and storage conditions with varying results.

Study	miRNAs	Species	Material	Storage Condition	Results	Reference
Enelund et al., 2017	miR-16, 23a, 26a, let7a	canine	serum, plasma, PAXGene	Storage for 24 h	PAXGene showed the highest values. Decline after 24 h in serum and plasma. There is no difference between serum and plasma.	[21]
Glinge et al., 2017	miR-1, 21, 29b	human	serum, plasma, EDTA, LiHep	Blood is stored for 4, 8, 12, 24 and 72 h before making serum 24 h and 9 months storage	There is no difference between EDTA, serum and plasma. EDTA storage has no effect until 72 h. Incubation of serum at RT showed reduction after 24 h.	[20]
Köberle et al., 2013	miR-1, 16, 21, 122, 142	human	serum	1-, 3-, 5-, and 24-h RT	Decline for miR-1 and 122. Slight decline for miR-16, 21, 142.	[16]
Kupec et al., 2022	miR-22, 23, 27b, 28, 99a, 100, 125b, 151a, 192, 193a, 193b, 194, 323b, 361, 1260a	human	serum	14 days at −80 °C	Slightly lower expression after storage. Differences between miRNAs.	[18]
Matias-Garcia et al., 2020	miR-23a, 24, 30c, 33b, 93, 103a, 124, 191, 451a	human	plasma	Long storage (17 years) at −80 °C	No effect.	[19]
McDonald et al., 2011	miR-15b, 16, 24	human	serum, plasma	24, 48, 72 h at 4 °C and −20 °C	Values are higher in plasma than in serum decline during storage.	[23]
Mitchell et al., 2008	miR-15b, 16, 19b, 24	human	serum, plasma	24 h at RT	There is no difference between serum and plasma. Minimal effect after 24 h RT.	[24]
Yamada et al., 2014	miR-16, 21, 29a, 125b	human	serum, plasma	7 days at 4 °C	More miR-16 in plasma than in serum. lower levels in stored sera for all.	[17]

**Table 2:** Signalment of the dogs included in the study.

No.	Breed	Age	Sex	Body Weight
1	Old German Herding Dog	5 years	fc	20 kg
2	Old German Herding Dog	5 years	f	20 kg
3	Tornjak	3 years	f	50 kg
4	Tornjak	3 years	mc	50 kg
5	Tibet Terrier	15 years	mc	11 kg
6	Cairn Terrier	6 years	fc	9 kg
7	German Shorthaired Pointer	1 year	m	26 kg
8	Mix	3 years	fc	12 kg
9	Australian Shepherd	2 years	f	23 kg
10	Mix	7 years	fc	12 kg

f = female, fc = female castrated, m = male, mc = male castrated.

**Table 3:** Copy number per µL of different miRNAs isolated from canine serum samples (*n* = 10) at time point 0 (about 2 h after sampling).

	Median c/µL	Min.–Max. c/µL
miR-20b	13,016	4781–26,041
miR-21	63,205	27,775–120,206
miR-122	919	231–2313
miR-126	17,331	4630–46,382
miR-192	7,298	3773–17,169
miR-214	5,160	1698–17,903
miR-222	15,063	5525–40,313
miR-494	556	27–965

## Data Availability

The raw data supporting the conclusions of this article will be made available by the authors on request.

## Abbreviations

The following abbreviations are used in this manuscript:

ddPCR	digital droplet PCR
miRNA	microRNA
miR	microRNA
